# Application and Perspectives of MALDI–TOF Mass Spectrometry in Clinical Microbiology Laboratories

**DOI:** 10.3390/microorganisms9071539

**Published:** 2021-07-20

**Authors:** Eva Torres-Sangiao, Cristina Leal Rodriguez, Carlos García-Riestra

**Affiliations:** 1 *Escherichia coli* Group, Foundation Institute of Health Research—University Hospital Complex of Santiago de Compostela (FIDIS—CHUS), ES-15706 Santiago de Compostela, Spain; cargarrie@gmail.com; 2 Clinical Microbiology and Parasitology Lab, University Hospital Marqués de Valdecilla (HUMV), ES-39008 Santander, Spain; 3 Novo Nordisk Foundation Center for Protein Research, Faculty of Health and Medical Sciences, University of Copenhagen, DK-2200 Copenhagen, Denmark; cristina.leal@cpr.ku.dk; 4 Clinical Microbiology and Parasitology Lab, University Hospital Complex of Santiago de Compostela (CHUS), ES-15706 Santiago de Compostela, Spain

**Keywords:** MALDI–TOF MS, microbial identification, proteomics, resistome, disease biomarkers

## Abstract

Early diagnosis of severe infections requires of a rapid and reliable diagnosis to initiate appropriate treatment, while avoiding unnecessary antimicrobial use and reducing associated morbidities and healthcare costs. It is a fact that conventional methods usually require more than 24–48 h to culture and profile bacterial species. Mass spectrometry (MS) is an analytical technique that has emerged as a powerful tool in clinical microbiology for identifying peptides and proteins, which makes it a promising tool for microbial identification. Matrix assisted laser desorption ionization–time of flight MS (MALDI–TOF MS) offers a cost- and time-effective alternative to conventional methods, such as bacterial culture and even 16S rRNA gene sequencing, for identifying viruses, bacteria and fungi and detecting virulence factors and mechanisms of resistance. This review provides an overview of the potential applications and perspectives of MS in clinical microbiology laboratories and proposes its use as a first-line method for microbial identification and diagnosis.

## 1. Background

Mass spectrometry (MS) was originally developed at the end of the 19th century to measure the masses of atoms, and one of its first contributions to science was demonstrating the existence of isotopes, at the beginning of the 20th century [1]. MS is an analytical approach that measures the mass-to-charge ratio (*m*/*z*) of chemical compounds and calculates its exact molecular weight. Laser desorption/ionization (LDI), matrix-assisted laser desorption ionization (MALDI) or surface-enhanced laser desorption/ionization (SELDI) as well as electrospray ionization (ESI) are currently the most widely used ionization techniques for analyzing chemical structures in biological systems [2]. In the late 1980s, with the introduction of soft ionization, protein analysis developed rapidly, revolutionizing MS. In the late 1990s, the pioneering application of MS in microbiology [1] demonstrated that intact bacterial cells could be distinguished using MALDI coupled to a time of flight (TOF) analyser [3]. These achievements stimulated the fast development of MALDI–TOF MS system approaches as promising tools for the microbial characterization of bacteria [4], fungi [5], viruses [6], and even nematodes [7].

**Figure 1:**
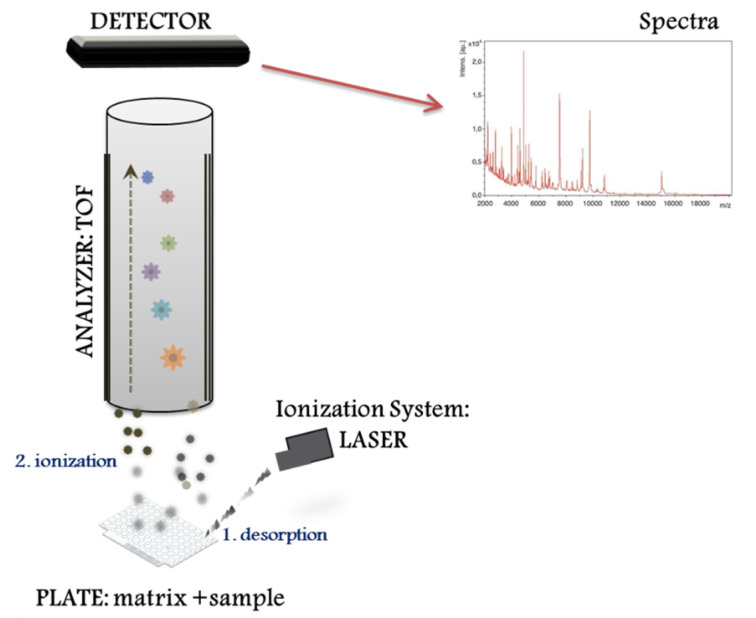
Schema showing the linear mode workflow in a MALDI–TOF MS system.

The MALDI–TOF MS system performs different proteomic strategies using intact or digested proteins. The “top down strategy” is used for direct analysis of intact proteins, proteoforms and post-translational protein modifications [8], whereas the “bottom up” is used for mixtures of peptides derived from protein digestion (i.e., peptide sequencing). MALDI–TOF MS systems represent a basic configuration workflow in a linear mode. Figure 1 presents a schematic overview divided into three compartments: (1) the ionization source system (laser), (2) the mass analyser (TOF), and (3) the ion detector. First, the sample for analysis is prepared by mixing with a matrix, an energy-absorbent, organic compound solution. Then, after the mixture (matrix and sample) crystallize upon drying, the sample is ionized using a laser beam. In the process of desorption and ionization using the ionization source system, the molecules are converted to gas-phase ions and individually charged [M+H]+, so that they can be manipulated by external electric and magnetic fields. MALDI is based on a soft ionization method that preserves the integrity of the sample without massive fragmentation [9]. Soft ionization allows the analysis of proteins and peptides and large organic molecules (i.e., polymers, dendrimers) [10], which tend to become brittle and fragmented in other ionization methods. Once the sample molecules are ionized, the ions are arranged and separated based on their *m*/*z* ratio using a mass analyser. For microbiological applications primarily, the TOF mass analyser is used. Last, an ion detection system (detector) measures the different separated ions and creates a mass spectrum, characterized by *m*/*z* ratios along with their relative abundance. As a result, the mass spectrum represents individual protein profiles (likewise a peptide mass fingerprints (PMF)) where different peaks correspond to different *m*/*z* ratios that can then be searched for protein mass values against a database containing known microbial isolates [11]. Therefore, the MALDI–TOF system identifies microorganisms using MS to determine a spectrum unique for each protein.

Microbial identification through MALDI–TOF MS presents multiple advantages. First, it is based on intact proteins, which avoids time-consuming digestions, desalting and other pre-treatments such as solid phase extraction while keeping good sensibility. Second, it has high transmission efficiency, fast scan rates and very low *m*/*z* ratio detection limits [9,12]. Third, its analysis is low cost, despite the initial investment in expensive equipment [13]. Because of these characteristics, MALDI–TOF MS has revolutionized clinical diagnosis, representing a fast and an effective method for the identification of bacterial profiles at both, the genus and species taxonomic levels. This new approach is being establishing as the preferred tool for identifying and characterizing microorganisms in microbiology laboratories, more so than even 16S and 18S rRNA gene sequencing [9,11,14,15].

The first main disadvantage is one relates to the need for trained laboratory personnel, beginning with the sample preparation [11]. The proper proportion between sample and matrix, and therefore the crystallization of the mixture, is an essential if massive fragmentation or even destruction of the sample is to be avoided. Moreover, bacterial age, the agar medium, bacteria culture atmosphere, the number of laser shots applied and the averaged spectra per measurement could interfere with the quality of the results and therefore to the proper identification of the sample [16]. The second disadvantage of MALDI–TOF MS is that the identification of new species relies strongly on a complete database [9].

In diagnostic microbiological laboratories, two functional MALDI–TOF systems analyze intact proteins: (1) microflex^®^ LT/SH MS or Biotyper (Bruker, Germany) and (2) VITEK^®^ MS (bioMérieux, France). In each case, the detection range of the TOF analyser is quite similar, but each relies on its own kits and databases [10,17]. Nonetheless, the identification accuracy of bacteria and fungi [18,19,20,21] is similarly good, but rather susceptible to sample preparation and culture media steps. In addition to the main advantages of simplicity and speed, the low analysis cost [13] has made MALDI–TOF MS a widely implemented diagnostic tool. Speed and reliability provide better patient prognosis and treatment, decreases hospitalization and reduces the risks associated with co-morbidity and mortality. In this review, we present an overview of the most recent advances in the field MALDI–TOF and proteomics focusing on applications for diagnosing infection.

## 2. Direct Microbial Identification from Human Samples

MALDI–TOF MS has recently been used in laboratories for the rapid identification of microorganisms in emergency and inpatients, resulting in shorter hospital stays, particularly in intensive care units [22]. Sepsis is a life-threatening organ dysfunction caused by an unregulated host response to infection [23] and is the major cause of mortality from infectious disease, according to Word Health Organization (https://www.who.int/news-room/fact-sheets/detail/sepsis, accessed on 26 August 2020). Bacterial meningitis is a neurological emergency. For both sepsis and meningitis, early diagnosis is vital for rapid initiation of antimicrobial treatment [24]. Direct detection and identification of microorganisms from blood and cerebrospinal fluid (CSF) are a relevant step.

Commercial protocols remain the reference procedures for the extraction of bacterial proteins from direct samples [10,25]. However, more and more modified and in-house methods have emerged to address several fundamental issues related to precision and speed of identification. As discussed in the previous section, sample preparation and an adequate method of protein extraction are key steps that may influence sensitivity, resolution, and reproducibility. Poor sample preparation will lead to lower peak resolution with a consequently lower sensitivity and reproducibility, since ion generation by MALDI–TOF depends on an optimal ratio of matrix substance and analyte [10]. Therefore, it is of the utmost importance to pretreat a sample properly considering that human fluids contain proteins other than bacterial or fungal. These preliminary steps must drain and separate blood and other human cells (i.e., haemoglobin), and selectively recover bacterial proteins [24]; otherwise, a wrong or unknown identification will result.

The direct identification of a microorganism in blood, CSF or urine is rather a matter of standardization because it requires a greater minimal bacterial load compared to other methods such as 16S rRNA gene sequencing or direct detection by Gram staining. Numerous studies have compared in-house methods although they have not shown any advantages over commercial ones. Most of them are based on the pre-treatment of samples by lysis, centrifugation, filtration and concentration, singly or in combination, or by the growth of monomicrobial cultures.

**Figure 2:**
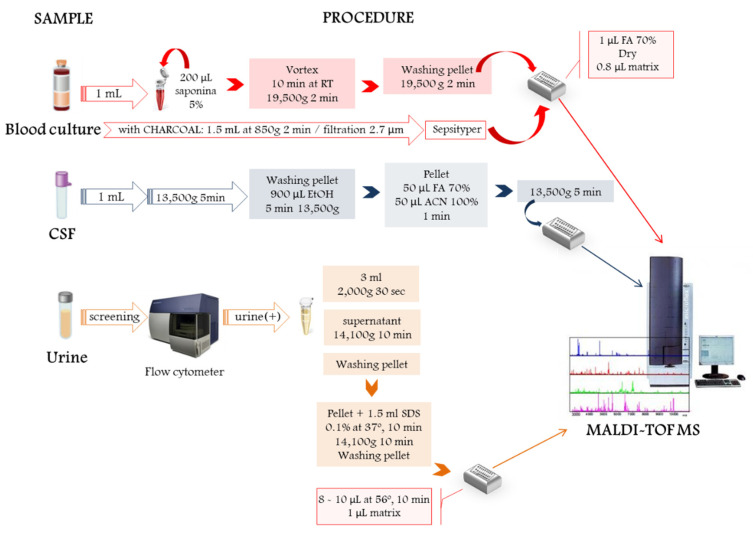
Hypothetical protein extraction workflow for direct microbiological diagnosis from biological samples from blood culture, cerebrospinal fluid (CSF) and urine.

Currently, the most common commercial protocols for blood culture are (1) the Sepsityper^®^ kit (Bruker Daltonics, Bremen, Germany) [26] extensively used and based on lysis and short centrifugation; (2) the VITEK^®^ MS blood culture kit (bioMérieux) [27,28], which collects bacteria using a filter; and (3) the rapid BACpro^®^ II kit (Nittobo Medical Co., Tokyo, Japan), which uses cationic particles to collect microorganisms in a relatively short runtime (approximately 15 min) [25,29,30]. On the other hand, modified in-house methods are also based on a previous lysis with saponin [31,32] or differential centrifugations of blood [33,34,35] prior to protein extraction to release and aid the separation of microorganisms and remove human proteins [17]. For example, Jakovljev and Berg [31] used a modified lysis method with 5% saponin, an additional pre-extraction, followed by protein extraction using 70% formic acid for best results [31]. The authors obtained identification levels of 99.3 and 96.6% with respect to genus and species, using low discrimination scores and a short processing period of only 25 min (Figure 2). The aim of all were to remove the multiple cells in blood samples that can interfere in the identification results.

The processing of CSF for the diagnosis of meningitis works in a similar way to urine samples (Figure 2) [10]. Before extraction with formic acid and acetonitrile, both are subjected to low-speed centrifugation to eliminate human cells [10,17,36]. For the processing of urine samples specifically, an initial flow cytometric scan is recommended to eliminate negative urine and limit direct processing to urine with counts greater than 10^5^ colony forming units per millilitre (CFU/mL) [17,24,37]. Recently, various research groups have proposed different extraction methods based on differential centrifugations [37,38], ultrafiltration [39], or a simple pre-treatment of samples with sodium-dodecyl-sulfate (SDS) [40,41]. A recent study conducted by Veron and colleagues [42] compared centrifugation (68.4% of correct MALDI identification), urine filtration (78.9%) and 5 h bacterial cultivation on solid culture media (84.2%), which demonstrated that a short culture step is the most straightforward and efficient sample preparation method for fast and reliable identification of uropathogens. However, none of the methods was able to improve the identification accuracy of microorganisms (<95%) or overcome the obstacle of the diagnosis of urinary infection linked to low colony counts (<10^4^ CFU/mL) or bi-/poly-microbial infections, though it is remarkable that the fast preliminary diagnosis took less than one hour.

Alternatively, a short incubation method on a solid medium was used by several clinical laboratories [43,44,45], although it did not produce any advances in the clinical diagnosis. Idelevich et al. [43], showed promising results with rapid identification at the species level in 1.2, 18.6, 64.0, 96.5, and 98.8% of Gram-positive cocci, and 76.2, 95.2, 97.6, 97.6, and 97.6% of Gram-negative rods, with incubation times from blood culture samples of  2, 4, 6, 8, and 12 h, respectively. Despite the success with Gram-negative and Gram-positive bacteria, direct identification of yeast and anaerobes failed, likely because both microorganisms require long incubation times (around 48 h). Despite the advances, a current challenge is the identification of microorganisms in polymicrobial cultures, representing a mixed bacterial fingerprint [46]. For this, short incubation methods have been used as well, but with low sensitivity (a low number of correctly microorganisms identified) [45]. There also exists a commercial module, Bruker ^®^MBT Sepsityper IVD, but it only identifies 34.3% of microorganisms correctly [47]. Thus, major improvements are still needed for polymicrobial blood cultures [48].

## 3. Microbial Identification Using Reference Databases and Open Free Libraries

Microorganism databases are the key component for the identification in MALDI–TOF MS. Proprietary databases (i.e., Bruker) are continuously updated and increasing in size as new microbial species are discovered and new annotations are made. In recent years, open database platforms, spectrum libraries and computer tools have emerged and been made available to the scientific community. Most of them are used to handling long sequences of peptide data [15]. Although, the conventional clinical microbiologist would find working with proprietary programs already in the operating system to be more practical, comfortable and familiar, open-source software platforms, in combination with proprietary ones such as OpenMS [49], allows for additional MS-based workflows and user interfaces that must be considered for clinical diagnoses. The standardization of protocols, the increase in the resources of spectrum libraries and databases, as well as the development of more intuitive computer programs, is allowing the MALDI–TOF system to consolidate itself as the method of choice for microbiological diagnosis [15] (Table 1).

There are two main platforms for commercial use: (1) MALDI BioTyper and (2) VITEK^®^ MS. Both provide a microbial diagnosis based on the detection and identification of low-molecular-weight spectra ranging from 2 to 20 KDa, typically represented by ribosomal proteins and a few housekeeping proteins [50]. They also show high levels of identification at the species level (S ~85%) [18,51] with MALDI BioTyper being better suited for bacteria and yeast [51] and VITEK^®^ MS (v3.0) being better suited for mycobacteria, actinomycetes [51,52,53] and filamentous fungi [54]. The MALDI BioTyper and VITEK^®^ MS platforms include software programs to explore and analyze the acquired mass spectra data. BioTyper and VITEK^®^ platforms are continuously updating their databases with discoveries of new specimens and annotations. These computer programs allow comparison, analysis of spectra clusters for strain classification [55], dendrogram performance, multidimensional analysis [56] and resistance determination using hydrolysis ratios [57,58]. The main difference and limitation between the platforms is in access to other networks or databases, and the resources of their respective computer programs. Despite having private access, BioTyper allows mass spectra and new identifications to be shared among all users.

The BioTyper system, conceived and marketed exclusively by Bruker Daltonics, uses its own database made up of more than 4000 entries (>3000 species of 540 genera). The proprietary database is open to users and allows the creation and exchange with other existing databases. Species identification is based on a numerical log score of identification and consistency, ranging from 0 to 3 [10,59]. A good species-level identification corresponds to values > 2.0, and a probable identification has a value between 2.30 and 3.0. At the genus level, a reliable score is between 1.70 and 2.29 [10,59], and not reliable if between 0.0 and 1.69 [10]. The MALDI BioTyper software also performs automatic calibration and creation of the main spectra of new clinical isolates [10]. The additional use of applications supporting interactive inspection and comparison of large datasets, such as ClinProTools, increases the precision of identification. For example, it can distinguish among *S. pneumoniae*, *S. oralis* and *S. mitis*, in the *Streptococcus viridans* group [60]; it can type *Streptococcus pyogenes* [61]; and it can distinguish between methicillin-sensitive *Staphylococcus aureus* (MSSA)^36^ and methicillin-resistant *S. aureus* (MRSA), as well as different MRSA clones [62,63]. Moreover, the BioTyper platform offers open libraries for enhanced accuracy identification (e.g., mycobacteria and filamentous fungi subtyping, or rapid and automatic calculation of intact and hydrolysed antibiotic substances (MBT STAR-BL application)).

The VITEK^®^ MS platform incorporates the SARAMIS database that uses SuperSpectra [10] as a reference to match and identify user query mass spectra. It is based on the mass spectra of at least 15 individual isolates [10] previously identified by 16S rRNA gene sequencing analysis or Multi-locus Sequencing typing (MLST). Microbial identification is based on the confidence interval (CI) percentage of the query mass spectra with respect to the SuperSpectra reference, interpreting a good identification at the species level when the CI is >95%, and not identifiable if it is not found in the database. Moreover, the Vitek MS v3.0 platform offers grouping of spectral data or hierarchical taxonomic analysis [10], permitting the identification of taxonomic changes in a population of microorganisms. For example, it allows for the successful differentiation between MRSA and MSSA through two MRSA marker peaks of (2305.6 and 3007.3 Da) and a single MSSA marker peak of (6816.7 Da) [64], or the discrimination between *Escherichia coli* ST131, with a sensitivity of 86.6% and a specificity of 95.1% [65].

Both MS systems offer a research-use-only (RUO) module that allows the creation of custom in-house libraries of mass spectra and provides workflow to detect of specific resistance mechanisms. The most immediate and relevant application is its use in clinical epidemiology, which is beginning to displace costly and laborious molecular methods such as MLST or Pulsed Field Gel Electrophoresis (PFGE) [17]. In addition, they both facilitate exportable and compatible mass spectral data, which generates more extensive data processing on external mathematical and statistical open-source platforms. The standardization of protocols, increase in the resources of the spectrum libraries and databases, and the development of more intuitive computer programs, is allowing the MALDI–TOF system to consolidate itself as the preferred method for microbiological diagnosis [15] (Table 1).

A recent advance is the new Bruker MALDI Biotyper^®^ Sirius which goes beyond microbial identification and is fully compatible with existing MALDI Biotyper^®^ software, libraries, and consumables. The MALDI–TOF MS used in positive-ion mode allows routine microbial identification, while the negative-ion mode broadens the microbial research applications such as lipid analysis. Lipidomics is a new “omic” that offers the rapid identification [66] of a mechanism of resistance, such as colistin resistance-related modifications to lipid A in colistin-resistant bacteria [67,68]. The next step is to improve the diagnosis of infections, using new libraries able to detect post-translational modifications (PTMs), human proteins or metabolites.

## 4. MS Big Data and Machine Learning Applied to Clinical Diagnosis

As previously discussed, the categorization, differentiation, and identification of microorganisms in MALDI–TOF MS relies strongly on spectrum libraries to identify peptide mass fingerprinting (PMF) generated from each microorganism. Even so, these databases are far from being complete and thus represent, in many cases, an important drawback because of missing or small-power PMF identifications. Alternatively, researchers have tried to increase the discriminatory power of PMF by increasing the number of biomarker peaks [14] using simple sonication techniques [69] up to short digestions with trypsin or pre-treatments with lysozyme [70]. Certainly, previous sample separations by, e.g., liquid chromatography (LC) provided more complete information on the sample’s composition [71], but it required qualified technical personnel and a longer processing time, which in turn resulted in a delayed diagnosis. Neither of these separation techniques has been successfully implemented in clinical microbiology laboratories.

Nonetheless, the increasing amounts of publicly available MS platforms in open access databases, mass spectrum libraries or analytical methods for visualization, standardization and validation [72,73] (Table 1), together with automated colony picking in the laboratory [24], are improving the typing or characterization of strains and identification of microbes. A starting point for a beginner in MS data analysis is presented by Chong and colleagues in their “Hitchiker’s guide” for MS bioinformatics analysis [74]. The use of open sources for bioinformatic platforms like Bioconductor (http://bioconductor.org/, accessed on 19 July 2021) are also a good starting point. There is also a wide range of mathematical and statistical programs that provide easy data handling: a mass spectral library, peak detection, processing, and alignment, as well as statistical analysis [75] (Table 1). For example, a quantification intensity count [76] is possible by generating reference mass spectra using the median intensity of the aligned peaks of all spectra or by calibration based on a total ion chromatogram (TIC) [76,77]. The semi-quantitative comparison of mass peaks (associated with specific proteins), then allows the comparison of a microorganism in different conditions, such as in urine and blood samples. Consequently, algorithms or mathematical models could be extrapolated to monitor infection and even detect resistance mechanisms early [58].

Other approaches are multidimensional analysis (MDS) or principal component analysis (PCA) (Table 1). MDS and PCA are mathematical approaches that use proximity measures such as the correlation coefficient or Euclidean distance to generate a spatial configuration of points in multidimensional space where distances between points reflect the similarity among isolates. MDS and PCA analyses have been extensively used to discover discriminative peaks [78] and identify potential sets of biomarkers [75,79] in a statistically reliable way [80], as well as distinguish among different strain isolates [79] from a large data set or selected genes or proteins [80,81].

## 5. The State-of-the-Art Combining Approaches

**Figure 3:**
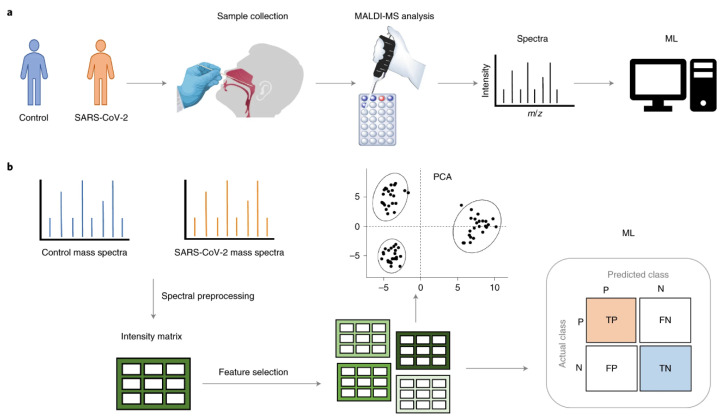
Representation of a standard MS workflow exemplified by SARS-CoV-2 detection in nasal mucous secretion. This figure was originally published in *Nature Biotechnology.* Nachtigall, F.M., Pereira, A., Trofymchuk, O.S. et al. Detection of SARS-CoV-2 in nasal swabs using MALDI-MS. *Nat. Biotechnol.* 38, 1168–1173 (2020). https://doi.org/10.1038/s41587-020-0644-7 ©Copyright Clearance Center. Reprint from [110].

Figure 3 presents a standard MS workflow with the exact identification of proteins and their relative quantification that provides detailed knowledge of protein expression, microorganisms and parasites, and their final integration and interaction in the human body.

In the recent years, MS has gained importance for characterizing nanoparticles, which has expanded the possibilities of MALDI–TOF MS [84,85,86] in the environmental sciences to study, for example, the distribution, concentration and stability of silver and gold nanoparticles in environmental water [87]. Research, using biological samples, are showing the reliable detection of sulfides, drugs and other substances [88] with good limits, especially for urine metabolites, e.g., cysteine and homocysteine with a detection range between 7 and 22 nanoMolar [89]. Chou et al. [90] have proposed a new clinical diagnostic approach using magnetic antibody nanoparticles for the rapid detection of influenza virus subtypes [90], while research led by Miotto [91] propose the use of maghemite nanoparticles for early diagnosis of mastitis in bovine milk.

Another MALDI–TOF MS combined approach currently used by few clinical microbiology laboratories is the Fourier Transform Infrared Spectroscopy (FTIRS), uses molecular vibration fingerprints, primarily the C–O stretching of biomacromolecules, to determine the molecular composition of a wide range of sample types [92]. By strain-specific absorbance patterns in the infrared spectrum [93], FTIRS characterizes a microbial sample by reflecting its biomolecular content to correlate with its genetic information [94]. FTIRS has been successfully applied in many studies to discriminate among bacteria at different taxonomic levels, (e.g., serogroup or serotype) and even at the strain level, to provide simple, quick, high-throughput, cost-effective bacterial typing [95,96,97]. The IR Biotyper (Bruker Daltonics, Bremen, Germany) is being used in the field of microbial strain typing, such as for the study of nosocomial outbreaks and their dynamics to prevent the spread of pathogens inside the hospitals [93].

During the last decade, MALDI–TOF has found application in biological systems [98] with the incorporation of imaging, the so called Mass Spectrometry Imaging or MALDI imaging [99]. It evolved rapidly and is commonly used in the diagnosis of inflammatory and infectious diseases in human or animal tissues, [100], including samples such as bacterial biofilm [101] or mammalians [102]. Imaging MS is being continuously updated and introducing new techniques for the diagnosis of disease [98], as well as for the diagnosis of infection. MS imaging could offer an especially useful diagnostic tool for skin and soft tissue infections as well as for the diagnosis of papillomavirus [103].

Last, polymerase chain reaction (PCR)-based MS was first described in 2011 by Yi et al. in the identification of human papillomavirus [104], and ever since most of the viral identification approaches have combined both techniques. On one hand, PCR is used for multiplexing and amplification, whereas MALDI–TOF is used to identify and analyse the amplified sample [15,105]. Therefore, PCR-based MS is an effective, low-cost tool for identifying various poliovirus serotypes [106], common respiratory viruses (CRV) [107] and the hepatovirus [15]. It can also be used for typing or subtyping influenza viruses [108] and even specific viral biomarkers to distinguish infected and healthy cells [106]. PCR-based MS also appears to be potentially effective in detecting resistance to drugs such as ganciclovir in transplant recipients infected with cytomegalovirus (CMV) [109].

## 6. Current Challenges: Viral Identification and Antimicrobial Resistance

### 6.1. Building Virus Spectral Libraries, Getting a Fast Diagnosis of SARS-2

Few studies have so far been conducted on identifying and detecting viruses partly due to the high molecular weight of viral proteins (>20 KDa) and because viral identification still relies strongly on cell culture and antigen or nucleic acid detection. In addition, extraction protocols for viral proteins frequently require of a previous step involving a cell substrate cultured in vitro, which could alter the proteins and take several days. Afterwards, amplification is used for a fast and sensitive diagnosis, and PCR is frequently the method of choice [106,111,112]. Other current limitations besides contamination and time relate to the low quantity of viral proteins in the biological sample, where human proteins are the most abundant, and to their rapid mutation, which complicates the design of specific spectral libraries [112].

A recent 2014 study from Calderaro et al. discriminated among three different serotypes of Sabin poliovirus, tuning the range of MALDI–TOF to identify differential PMF peaks for the VP4 capsid [106]. Two years later, the same research group created a main spectrum profile (MSP) to identify CRVs, including influenza A and B; adenovirus C; parainfluenza types 1, 2, and 3; respiratory syncytial virus (RSV); echovirus; CMV; and human metapneumovirus. The MSP was based on the mass spectra of infected samples (differential peaks) that had previously been compared with uninfected cells [113].

At present with the current COVID-19 pandemic, the need for a rapid diagnosis of SARS-CoV-2 has generated a rapid evolution and optimization of clinical methods, including MALDI–TOF MS. Because the gold standard and main diagnosis tool for SARS-CoV-2, the RT-PCR, is continuously producing false negatives, questions are being raised about whether MS-based technology, such as MassARRAY, which demonstrates superior sensitivity and discrimination of mutations within the viral genome, should overtake it [114,115]. MS-based methods using swab and saliva samples are reporting promising results. Illes RK et al. [116] achieved a multifaceted clinical MALDI–TOF MS screening test, primarily (but not limited to) SARS-CoV-2 by detecting viral envelope glycoproteins, including peaks of spike protein fragments S1, S2b, and S2a. The method offers ease of sampling, speed of analysis, and a much lower cost of testing.

Another proof of concept is the combination of MS-based methods with machine learning (ML) and artificial intelligence (AI) [117], which is also demonstrating reliable detection of SARS-CoV-2 in swab samples. Tran et al. [118] evaluated an automated ML platform, Machine Intelligence Learning Optimizer (MILO), combined with MALDI–TOF MS for rapid high-throughput screening of COVID-19 and showing promising accuracy (96.6–98.3%), sensitivity (positive percent agreement of 98.5–100%), and specificity (negative percent agreement of 94–96%) [118], respectively, for two different ML models. Similarly, Delofeu et al. analyzed 236 nasopharyngeal swab samples, and the subsequent mass spectra data was used to build different ML models, showing a performance of >90% accuracy, sensitivity, and specificity. They compared extreme gradient boosting trees and support vector machines (SVMs), and the best results were obtained from an SVM. As a last example, research by Nachtigall et al. [110] evaluated six different ML models that demonstrated high accuracy and reliability with the highest accuracy (93.9%) presented by a SVM, with 7% false positives and 5% false negatives (Figure 3).

### 6.2. Identifying Mechanisms of Resistance

Another critical responsibility for MALDI–TOF MS in clinical microbiological diagnosis is in determining resistance mechanisms. Currently, these methods require long and laborious incubations and hydrolysis protocols (1–4 h) [10,119] with no greater advantages compared to rapid agglutination, immunochromatographic methods (15–30 min) or automated phenotypic tests [10,120]. Some authors advocated the use of selective extraction methods to recover cell wall components [15] or periplasmic and membrane proteins (commonly involved in more than half of bacterial resistance mechanisms), in addition to using different matrices to detect them [15,119]. On the other hand, the construction of large spectra libraries with bacterial resistant PMF would be a simple alternative that would allow comparative studies into the role of various components involved in antibiotic resistance [119].

The detection of antibiotic resistance in parallel with bacterial identification is the unreachable goal in a routine diagnosis. As is the case with viral identification, most bacterial proteins involved in resistance as well as virulence have a molecular weight > 20 KDa, which puts it out of the usual range of the clinical use of MALDI–TOF MS. However, software such as MBT-STAR-LAB, provided by Bruker, facilitates the detection of for example, carbapenem-resistant isolates by identifying carbapenem hydrolysis identification peaks, not the protein enzyme. Another program, CinProTools (Bruker Daltonics, Germany) [35], detects MRSA by overlapping mass spectra to characterize a specific common peak. For example, MRSA-detecting peptides, such as phenol-soluble modulin (PSM-mec), which are linked to the class A *mec* gene complex, are present in MRSA strain SCCmec cassette types III and VIII, and are automatically interpreted by the MBT Subtyping Module. However, any of these proposed methods is time-consuming and might delay diagnosis by at least 24 h.

Nix et al. developed a satisfactory method to identify bacteria and resistance simultaneously. Briefly, the authors incubated the samples directly from positive blood cultures at different antibiotic concentrations on the surface of the MALDI target for 4 h at 37 °C in a humidity chamber. Afterwards the medium was removed and an on-target protein extraction was performed with formic acid before adding a matrix with an internal standard as a quality control [121]. The robustness and reproducibility of this direct-on-target microdroplet growth assay (DOT–MGA) proved to be a rapid and accurate identification tool applicable to a broad range of microorganisms and antimicrobial resistance. It also demonstrated its capacity for automation, especially for determining clinical samples directly [122,123]. The main limitations were standard assay conditions, such as humidity, medium removal, MALDI–TOF MS settings, internal control, and spectra analysis [124].

## 7. An MS-Systems Biology Approach: The Future of Identification

Until now, clinical development has been directly adopted from proteomic or interatomic studies, with additional extensive sample preparation steps (e.g., binding, DIGE, isobaric labeling, LC, 2D-LC, and nanoLC). Although it is difficult to predict how MS-based approaches will develop, the future is exciting, especially in the area of life sciences relating to viruses, host-pathogen interactions and vaccines. Along with the evolution of system biology, MS is expected to improve understanding of how viruses enter cells, how bacteria and virus defeat immunological barriers and which processes arrest cellular metabolism [113]. Systems biology is becoming a major component for understanding infection, due to its interdisciplinary nature and association with high-performance techniques. Moreover, data management of these systems, like any other field [125], can easily be implemented in microbiological diagnostic laboratories [126]. Even though the promises of systems biology are many [127], the underlying molecular mechanisms [127] must be experimentally verified using other approaches, such as transcriptomics or proteomics [126].

**Figure 4:**
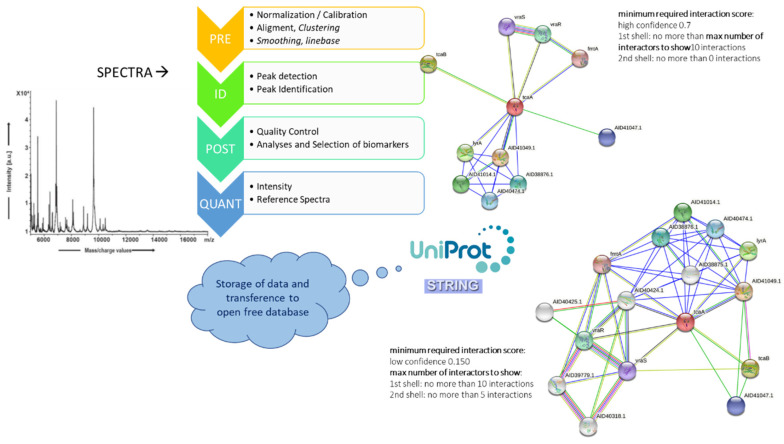
A possible computational workflow for data analysis from MALDI–TOF MS using the STRING database. Example of a network of interactions for the TcaA protein involved in *Staphylococcus aureus* resistance to glycopeptides using STRING. The membrane-associated protein is co-expressed upstream with the peripheral membrane protein FmtA which affects the methicillin resistance. TcaAB is responsible for susceptibility to glycopeptides, especially teicoplanin, in *S. aureus* [138,139]. The dysfunction or absence of either of these two proteins TcaAB is associated with resistance to glycopeptides and is subject to regulation by the two-component system VraSR (S: sensor, R: regulator) [138,140].

Currently, a wide variety of bioinformatics platforms have been developed that allow the analysis and visualization of protein–protein interaction networks in which interactive diagrams where nodes represent the proteins, and links represent the interactions [126]. Some platforms constitute only a few microorganisms, such as *E. coli*, and more recently *S. aureus* (Table 2), but there exist also other platforms having an extensive number of microorganisms, such as STRING one of the most extensive database [128]. STRING provides a simple graphic interface and easy interaction navigation as well as the ability to export of graphs and coordinates. Protein–protein networks are useful for revealing interactions between virulence factors or resistance proteins (typically with high molecular weight) with proteins of lower molecular weight detectable by MALDI–TOF MS (Figure 4).

The major limitations of applying a system-biology approach in clinical laboratories are the poor reproducibility and the lack of external validation, which impede progress in refining the identification of microbial species and streamlining antimicrobial resistance profiling. Open-access platforms that share workflows, and databases that rely on growth conditions (medium, time or atmosphere), are needed to optimize network information.

## 8. Conclusions

More than a decade after the arrival of MALDI–TOF MS in clinical microbiology laboratories, MALDI has become among the most conventional molecular methods of for microbiological diagnosis. Part of this success can be attributed to its speed, reliability, and low cost. Nonetheless, traditional bacteriological methods like quality control of clinical samples, Gram-staining results or colony growth observations are still important because they are the first and confirmatory diagnoses, respectively.

The MALDI–TOF system represents an important technique not only for microbial identification, but also for the epidemiological control of virulent strains and possible outbreaks or emergencies, in which rapid identification leads to a rapid response that ensures the control of the emergency. Future combinations with other systems or approaches, such as system biology offer a reliable quick and reliable diagnosis, but there is still much to do to standardize methods and analyses.

The evolution of pulse ionization techniques, together with the rapid evolution of improved-resolution infrastructure and instrumentation will open the door for a powerful third-generation MALDI–TOF MS with better peak quality, higher mass accuracy, and greater identification sensitivity. The next step in microbiological diagnosis could even be the protein sequencing of microorganisms by tandem MSs.

## 9. Websites

1. BioNumerics: http://www.applied-maths.com/, accessed on 19 July 2021
2. BRUKER BioTyper: https://www.bruker.com/es/service/support-upgrades/software-downloads/mass-spectrometry.html, accessed on 19 July 2021
3. Institute for System Biology: https://www.systemsbiology.org/, accessed on 19 July 2021
4. MATLAB: http://es.mathworks.com/products/matlab/, accessed on 19 July 2021
5. Matrix Science: http://www.matrixscience.com/, accessed on 19 July 2021
6. Seattle Proteome Center: http://www.proteomecenter.org/, accessed on 19 July 2021
7. Swiss Institute of BioInformatics: http://www.isb-sib.ch/, accessed on 19 July 2021
8. The European Bioinformatics Institute (EMBL-EBI): http://www.ebi.ac.uk/, accessed on 19 July 2021
9. UniProt: http://www.uniprot.org/, accessed on 19 July 2021
10. VITEK-MS: http://www.vitekms.com/, accessed on 19 July 2021

## Figures and Tables

**Table 1:** Overview of platforms for the analysis of big MS data of acquired mass spectra from MALDI–TOF MS. MDS: Multi-dimensional Scaling; PCA: principal component analysis; QA/QC: Quality Assurance/Quality Control; SOM: self-organization Map.

Platform	Statistical Analysis	Spectra Analysis	Accepted Formats	URL	Reference
BioNumerics	ANOVA, MANOVA, PCA, MDS, SOM and other statistical, parametric and non-parametric tests. Dendograms, cluster analysis, bioclustering, generation of phylogenetic trees. QA/QC.	Creation, identification and classification (spectrum libraries). Pre-processing: optimization, normalization, alignment, subtraction, smoothing. Peak detection, identification and quantification.	mzML, *.btmsp, *.txt RAW	https://www.applied-maths.com/applications/maldi-tof-bacterial-identification (accessed on 19 July 2021)	Bionumerics™ software (Applied Maths BVBA, Sint-Martens-Lantem, Belgium).
MaldiQUANT	Computational framework in R language: statistical analysis, dendograms, clustering, probability distributions, quality control, etc.	Pre-processing: optimization, normalization, alignment, subtraction, smoothing. Peak detection, identification, and quantification.	mzML, mzXLM, imzML *.csv, *.fid, *.tab	http://strimmerlab.org/software/maldiquant/ (accessed on 19 July 2021)	Gibb S and Strimmer [82]
Mass-Up	PCA, classification analysis, biomarker discovery, clustering and bioclustering. QA/QC.	Preprocessing: intensity transformation, optimization, alignment, subtraction, smoothing and peak analysis. Peak detection and identification.	mzML, mzXLM, *.csv, *.muc	http://www.sing-group.org/mass-up/ (accessed on 19 July 2021)	López-Fernandez et al. [83]
MATLAB	Regression, ANOVA, PCA, multivariate analysis, probability distributions, cluster analysis.	Pre-processing: optimization, smoothing, alignment, signal statistics, peak analysis, envelope extraction. Spectral analysis.	*.txt, *.xls, *.xlsx	http://es.mathworks.com/products/matlab-online/ (accessed on 19 July 2021)	MATLAB^®^ software (MathWorks Inc., Natick, MA, USA)
PEAKS	Algorithms and support for analysis.	Pre-processing: optimization, normalization, alignment, subtraction, smoothing, peak analysis. Peak detection, identification and quantification. Sequence editor.	mzML, mzXLM, mzDATA, MGF, ASCII	http://www.bioinfor.com/ (accessed on 19 July 2021)	Peaks^®^ software (Bioinformatics Solutions Inc., Waterloo, ON, Canada)

**Table 2:** Overview of platforms with application for protein analysis.

Platform	Database	What Is	Applications	URL	Reference
AureoLib/Aurewiki	*Staphylococcus. aureus*	AureoLib is a library to provide easy and intuitive access to protein synthesis data derived from various proteomics experiments	Aureolib provides protein synthesis data derived from various proteomic experiments (adaptation processes); Aurewiki provides functional and expression data (pangenome).	http://www.aureolib.de (accessed on 19 July 2021)	Fuchs et al. [129]
BioGrid	Multiplex	The Biological General Repository for Interaction Datasets (BioGRID) is a public database focused on specific biological processes with disease relevance.	Repository of genetic and protein interactions, chemical associations, and post-translational modifications, from model organisms and humans.	http://thebiogrid.org/ (accessed on 19 July 2021)	Oughtred et al. [130,131]
Cytoscape	Multiplex	Cytoscape is an open source software platform	Visualization of the molecular interaction networks and biological pathways, and integration of these networks with annotations, gene expression profiles and other data.	http://www.cytoscape.org/index.html (accessed on 19 July 2021)	Shannon et al. [132], Smoot ME et al. [133]
PeptideShaker	Multiplex	PeptideShaker is a search engine platform for the identification of proteins from multiple searches and *de novo* engines.	Protein identification with functional data.	http://compomics.github.io/projects/peptide-shaker.html (accessed on 19 July 2021)	Vaudel et al. [134]
PeptideAtlas	Multiplex	PeptideAtlas is a multi-organism, publicly accessible compendium of peptides identified in a large set of tandem MS proteomics experiments.	Protein identification, full annotation, database, data repository, peptides sequence, mapping and storing among others	http://www.peptideatlas.org/ (accessed on 19 July 2021)	Desiere et al. [135]
REACTOME	Multiplex	REACTOME is an open-access, manually curated and peer-reviewed pathway database.	Visualization, interpretation and analysis of pathways and interactions in the human biological system.	http://www.reactome.org/ (accessed on 19 July 2021)	Fragegat et al. [136]
STRING	Multiplex	STRING is a database of well-known protein interactions, including direct (physical) and indirect (functional) associations, aggregated from other (primary) databases.	Protein–protein interaction identification, pathway analysis, network connectivity, functional prioritazation.	https://string-db.org/ (accessed on 19 July 2021)	Snel et al. [137] Szklarczyk et al. [128]

## Data Availability

Not applicable.

## References

[1] Griffiths J. (2008). A brief history of mass spectrometry. Anal. Chem..

[2] Fenn J.B., Mann M., Meng C.K., Wong S.F., Whitehouse C.M. (1989). Electrospray ionization for mass spectrometry of large biomolecules. Science.

[3] Holland R.D., Wilkes J.G., Rafii F., Sutherland J.B., Persons C.C., Voorhees K.J., Lay J.O. (1996). Rapid identification of intact whole bacteria based on spectral patterns using matrix-assisted laser desorption/ionization with time-of-flight mass spectrometry. Rapid Commun. Mass Spectrom. RCM.

[4] Dieckmann R., Helmuth R., Erhard M., Malorny B. (2008). Rapid classification and identification of salmonellae at the species and subspecies levels by whole-cell matrix-assisted laser desorption ionization-time of flight mass spectrometry. Appl. Environ. Microbiol..

[5] Valentine N.B., Wahl J.H., Kingsley M.T., Wahl K.L. (2002). Direct surface analysis of fungal species by matrix-assisted laser desorption/ionization mass spectrometry. Rapid Commun. Mass Spectrom. RCM.

[6] Colquhoun D.R., Schwab K.J., Cole R.N., Halden R.U. (2006). Detection of norovirus capsid protein in authentic standards and in stool extracts by matrix-assisted laser desorption ionization and nanospray mass spectrometry. Appl. Environ. Microbiol..

[7] Perera M.R., Vanstone V.A., Jones M.G. (2005). A novel approach to identify plant parasitic nematodes using matrix-assisted laser desorption/ionization time-of-flight mass spectrometry. Rapid Commun. Mass Spectrom. RCM.

[8] Gregorich Z.R., Ge Y. (2014). Top-down proteomics in health and disease: Challenges and opportunities. Proteomics.

[9] Altun O., Botero-Kleiven S., Carlsson S., Ullberg M., Ozenci V. (2015). Rapid identification of bacteria from positive blood culture bottles by MALDI-TOF MS following short-term incubation on solid media. J. Med. Microbiol..

[10] Clark A.E., Kaleta E.J., Arora A., Wolk D.M. (2013). Matrix-assisted laser desorption ionization-time of flight mass spectrometry: A fundamental shift in the routine practice of clinical microbiology. Clin. Microbiol. Rev..

[11] Greco V., Piras C., Pieroni L., Ronci M., Putignani L., Roncada P., Urbani A. (2018). Applications of MALDI-TOF mass spectrometry in clinical proteomics. Expert. Rev. Proteom..

[12] Abián J., Carrascal M., Gay M. (2008). . Introducción a la Espectrometría de Masas Para la Caracterización de Péptidos y Proteínas en Proteómica.

[13] Fagerquist C.K., Garbus B.R., Miller W.G., Williams K.E., Yee E., Bates A.H., Boyle S., Harden L.A., Cooley M.B., Mandrell R.E. (2010). Rapid identification of protein biomarkers of Escherichia coli O157:H7 by matrix-assisted laser desorption ionization-time-of-flight-time-of-flight mass spectrometry and top-down proteomics. Anal. Chem..

[14] Sandrin T.R., Goldstein J.E., Schumaker S. (2013). MALDI TOF MS profiling of bacteria at the strain level: A review. Mass Spectrom. Rev..

[15] Singhal N., Kumar M., Kanaujia P.K., Virdi J.S. (2015). MALDI-TOF mass spectrometry: An emerging technology for microbial identification and diagnosis. Front. Microbiol..

[16] Cuenod A., Foucault F., Pfluger V., Egli A. (2021). Factors Associated With MALDI-TOF Mass Spectral Quality of Species Identification in Clinical Routine Diagnostics. Front. Cell Infect. Microbiol..

[17] De Carolis E., Vella A., Vaccaro L., Torelli R., Spanu T., Fiori B., Posteraro B., Sanguinetti M. (2014). Application of MALDI-TOF mass spectrometry in clinical diagnostic microbiology. J. Infect. Dev. Ctries.

[18] Jamal W.Y., Ahmad S., Khan Z.U., Rotimi V.O. (2014). Comparative evaluation of two matrix-assisted laser desorption/ionization time-of-flight mass spectrometry (MALDI-TOF MS) systems for the identification of clinically significant yeasts. Int. J. Infect. Dis..

[19] Deak E., Charlton C.L., Bobenchik A.M., Miller S.A., Pollett S., McHardy I.H., Wu M.T., Garner O.B. (2015). Comparison of the Vitek MS and Bruker Microflex LT MALDI-TOF MS platforms for routine identification of commonly isolated bacteria and yeast in the clinical microbiology laboratory. Diagn. Microbiol. Infect. Dis..

[20] Lavergne R.A., Chauvin P., Valentin A., Fillaux J., Roques-Malecaze C., Arnaud S., Menard S., Magnaval J.F., Berry A., Cassaing S. (2013). An extraction method of positive blood cultures for direct identification of Candida species by Vitek MS matrix-assisted laser desorption ionization time of flight mass spectrometry. Med. Mycol..

[21] Teke L., Barış A., Bayraktar B. (2021). Comparative evaluation of the Bruker Biotyper and Vitek MS matrix-assisted laser desorption ionization-time of flight mass spectrometry (MALDI-TOF MS) systems for non-albicans Candida and uncommon yeast isolates. J. Microbiol. Methods.

[22] Ge M.-C., Kuo A.-J., Liu K.-L., Wen Y.-H., Chia J.-H., Chang P.-Y., Lee M.-H., Wu T.-L., Chang S.-C., Lu J.-J. (2017). Routine identification of microorganisms by matrix-assisted laser desorption ionization time-of-flight mass spectrometry: Success rate, economic analysis, and clinical outcome. J. Microbiol. Immunol. Infect..

[23] Singer M., Deutschman C.S., Seymour C.W., Shankar-Hari M., Annane D., Bauer M., Bellomo R., Bernard G.R., Chiche J.-D., Coopersmith C.M. (2016). The Third International Consensus Definitions for Sepsis and Septic Shock (Sepsis-3). JAMA.

[24] Tsuchida S., Umemura H., Nakayama T. (2020). Current Status of Matrix-Assisted Laser Desorption/Ionization-Time-of-Flight Mass Spectrometry (MALDI-TOF MS) in Clinical Diagnostic Microbiology. Molecules.

[25] Nomura F., Tsuchida S., Murata S., Satoh M., Matsushita K. (2020). Mass spectrometry-based microbiological testing for blood stream infection. Clin. Proteom..

[26] Schubert S., Weinert K., Wagner C., Gunzl B., Wieser A., Maier T., Kostrzewa M. (2011). Novel, improved sample preparation for rapid, direct identification from positive blood cultures using matrix-assisted laser desorption/ionization time-of-flight (MALDI-TOF) mass spectrometry. J. Mol. Diagn..

[27] Fothergill A., Kasinathan V., Hyman J., Walsh J., Drake T., Wang Y.F. (2013). Rapid identification of bacteria and yeasts from positive-blood-culture bottles by using a lysis-filtration method and matrix-assisted laser desorption ionization-time of flight mass spectrum analysis with the SARAMIS database. J. Clin. Microbiol..

[28] Broyer P., Perrot N., Rostaing H., Blaze J., Pinston F., Gervasi G., Charles M.H., Dachaud F., Dachaud J., Moulin F. (2018). An Automated Sample Preparation Instrument to Accelerate Positive Blood Cultures Microbial Identification by MALDI-TOF Mass Spectrometry (Vitek((R))MS). Front. Microbiol..

[29] Ashizawa K., Murata S., Terada T., Ito D., Bunya M., Watanabe K., Teruuchi Y., Tsuchida S., Satoh M., Nishimura M. (2017). Applications of copolymer for rapid identification of bacteria in blood culture broths using matrix-assisted laser desorption ionization time-of-flight mass spectrometry. J. Microbiol. Methods.

[30] Tsuchida S., Murata S., Miyabe A., Satoh M., Takiwaki M., Ashizawa K., Terada T., Ito D., Matsushita K., Nomura F. (2018). Application of the biocopolymer preparation system, rapid BACpro^®^ II kit, for mass-spectrometry-based bacterial identification from positive blood culture bottles by the MALDI Biotyper system. J. Microbiol. Methods.

[31] Jakovljev A., Bergh K. (2015). Development of a rapid and simplified protocol for direct bacterial identification from positive blood cultures by using matrix assisted laser desorption ionization time-of- flight mass spectrometry. BMC Microbiol..

[32] Chen J.H., Ho P.L., Kwan G.S., She K.K., Siu G.K., Cheng V.C., Yuen K.Y., Yam W.C. (2013). Direct bacterial identification in positive blood cultures by use of two commercial matrix-assisted laser desorption ionization-time of flight mass spectrometry systems. J. Clin. Microbiol..

[33] Riederer K., Cruz K., Shemes S., Szpunar S., Fishbain J.T. (2015). MALDI-TOF identification of Gram-negative bacteria directly from blood culture bottles containing charcoal: Sepsityper(R) kits versus centrifugation-filtration method. Diagn. Microbiol. Infect. Dis..

[34] Gray T.J., Thomas L., Olma T., Iredell J.R., Chen S.C. (2013). Rapid identification of Gram-negative organisms from blood culture bottles using a modified extraction method and MALDI-TOF mass spectrometry. Diagn. Microbiol. Infect. Dis..

[35] Idelevich E.A., Storck L.M., Sparbier K., Drews O., Kostrzewa M., Becker K. (2018). Rapid Direct Susceptibility Testing from Positive Blood Cultures by the Matrix-Assisted Laser Desorption Ionization-Time of Flight Mass Spectrometry-Based Direct-on-Target Microdroplet Growth Assay. J. Clin. Microbiol..

[36] Bishop B., Geffen Y., Plaut A., Kassis O., Bitterman R., Paul M., Neuberger A. (2018). The use of matrix-assisted laser desorption/ionization time-of-flight mass spectrometry for rapid bacterial identification in patients with smear-positive bacterial meningitis. Clin. Microbiol. Infect..

[37] Zboromyrska Y., Bosch J., Aramburu J., Cuadros J., Garcia-Riestra C., Guzman-Puche J., Liebana Martos C., Loza E., Munoz-Algarra M., Ruiz de Alegria C. (2018). A multicentre study investigating parameters which influence direct bacterial identification from urine. PLoS ONE.

[38] Ferreira L., Sanchez-Juanes F., Munoz-Bellido J.L., Gonzalez-Buitrago J.M. (2011). Rapid method for direct identification of bacteria in urine and blood culture samples by matrix-assisted laser desorption ionization time-of-flight mass spectrometry: Intact cell vs. extraction method. Clin. Microbiol. Infect..

[39] Demarco M.L., Burnham C.A. (2014). Diafiltration MALDI-TOF mass spectrometry method for culture-independent detection and identification of pathogens directly from urine specimens. Am. J. Clin. Pathol..

[40] Sanchez-Juanes F., Siller Ruiz M., Moreno Obregon F., Criado Gonzalez M., Hernandez Egido S., de Frutos Serna M., Gonzalez-Buitrago J.M., Munoz-Bellido J.L. (2014). Pretreatment of urine samples with SDS improves direct identification of urinary tract pathogens with matrix-assisted laser desorption ionization-time of flight mass spectrometry. J. Clin. Microbiol..

[41] Kim Y., Park K.G., Lee K., Park Y.J. (2015). Direct Identification of Urinary Tract Pathogens From Urine Samples Using the Vitek MS System Based on Matrix-Assisted Laser Desorption Ionization-Time of Flight Mass Spectrometry. Ann. Lab. Med..

[42] Veron L., Mailler S., Girard V., Muller B.H., L’Hostis G., Ducruix C., Lesenne A., Richez A., Rostaing H., Lanet V. (2015). Rapid urine preparation prior to identification of uropathogens by MALDI-TOF MS. Eur. J. Clin. Microbiol. Infect. Dis..

[43] Idelevich E.A., Schule I., Grunastel B., Wullenweber J., Peters G., Becker K. (2014). Rapid identification of microorganisms from positive blood cultures by MALDI-TOF mass spectrometry subsequent to very short-term incubation on solid medium. Clin. Microbiol. Infect..

[44] Ha J., Hong S.K., Han G.H., Kim M., Yong D., Lee K. (2018). Same-Day Identification and Antimicrobial Susceptibility Testing of Bacteria in Positive Blood Culture Broths Using Short-Term Incubation on Solid Medium with the MicroFlex LT, Vitek-MS, and Vitek2 Systems. Ann. Lab. Med..

[45] Florio W., Cappellini S., Giordano C., Vecchione A., Ghelardi E., Lupetti A. (2019). A new culture-based method for rapid identification of microorganisms in polymicrobial blood cultures by MALDI-TOF MS. BMC Microbiol..

[46] Royo-Cebrecos C., Gudiol C., Ardanuy C., Pomares H., Calvo M., Carratalà J. (2017). A fresh look at polymicrobial bloodstream infection in cancer patients. PLoS ONE.

[47] Scohy A., Noël A., Boeras A., Brassinne L., Laurent T., Rodriguez-Villalobos H., Verroken A. (2018). Evaluation of the Bruker^®^ MBT Sepsityper IVD module for the identification of polymicrobial blood cultures with MALDI-TOF MS. Eur. J. Clin. Microbiol. Infect. Dis..

[48] Faron M.L., Buchan B.W., Ledeboer N.A. (2017). Matrix-Assisted Laser Desorption Ionization-Time of Flight Mass Spectrometry for Use with Positive Blood Cultures: Methodology, Performance, and Optimization. J. Clin. Microbiol..

[49] Röst H.L., Sachsenberg T., Aiche S., Bielow C., Weisser H., Aicheler F., Andreotti S., Ehrlich H.-C., Gutenbrunner P., Kenar E. (2016). OpenMS: A flexible open-source software platform for mass spectrometry data analysis. Nat. Methods.

[50] Croxatto A., Prod’hom G., Greub G. (2012). Applications of MALDI-TOF mass spectrometry in clinical diagnostic microbiology. FEMS Microbiol. Rev..

[51] Levesque S., Dufresne P.J., Soualhine H., Domingo M.C., Bekal S., Lefebvre B., Tremblay C. (2015). A Side by Side Comparison of Bruker Biotyper and VITEK MS: Utility of MALDI-TOF MS Technology for Microorganism Identification in a Public Health Reference Laboratory. PLoS ONE.

[52] Wilen C.B., McMullen A.R., Burnham C.A. (2015). Comparison of Sample Preparation Methods, Instrumentation Platforms, and Contemporary Commercial Databases for Identification of Clinically Relevant Mycobacteria by Matrix-Assisted Laser Desorption Ionization-Time of Flight Mass Spectrometry. J. Clin. Microbiol..

[53] Brown-Elliott B.A., Fritsche T.R., Olson B.J., Vasireddy S., Vasireddy R., Iakhiaeva E., Alame D., Wallace R.J., Branda J.A. (2019). Comparison of Two Commercial Matrix-Assisted Laser Desorption/Ionization-Time of Flight Mass Spectrometry (MALDI-TOF MS) Systems for Identification of Nontuberculous Mycobacteria. Am. J. Clin. Pathol..

[54] Sun Y., Guo J., Chen R., Hu L., Xia Q., Wu W., Wang J., Hu F. (2020). Multicenter evaluation of three different MALDI-TOF MS systems for identification of clinically relevant filamentous fungi. Med. Mycol..

[55] Camoez M., Sierra J.M., Dominguez M.A., Ferrer-Navarro M., Vila J., Roca I. (2015). Automated categorization of methicillin-resistant Staphylococcus aureus clinical isolates into different clonal complexes by MALDI-TOF mass spectrometry. Clin. Microbiol. Infect..

[56] Deol P., Girard V., Hyman J., Miller E., Dussoulier R., Mailler S., Schrenzel J., Beni A.M., Ninet Bescher B., Walsh J., BioMerieux (2013). Identification of Mycobacteria by VITEK^®^ MS MatrixAssisted Laser Desorption Ionization—Time of Flight Mass Spectrometry.

[57] Mirande C., Canard I., Perrot N., Welker M., Van Belkum A., Chatellier S., BioMerieux (2013). Matrix-Assisted Laser Desorption Ionization—Time of Flight Mass Spectrometry for Rapid Antibiotic Resistance Detection.

[58] Angeletti S., Dicuonzo G., Lo Presti A., Cella E., Crea F., Avola A., Vitali M.A., Fagioni M., De Florio L. (2015). MALDI-TOF mass spectrometry and blakpc gene phylogenetic analysis of an outbreak of carbapenem-resistant K. pneumoniae strains. New Microbiol..

[59] Morgenthaler N.G., Kostrzewa M. (2015). Rapid identification of pathogens in positive blood culture of patients with sepsis: Review and meta-analysis of the performance of the sepsityper kit. Int. J. Microbiol..

[60] Chen J.H., She K.K., Wong O.Y., Teng J.L., Yam W.C., Lau S.K., Woo P.C., Cheng V.C., Yuen K.Y. (2015). Use of MALDI Biotyper plus ClinProTools mass spectra analysis for correct identification of Streptococcus pneumoniae and Streptococcus mitis/oralis. J. Clin. Pathol..

[61] Tadros M., Cabrera A., Matukas L.M., Muller M. (2019). Evaluation of Matrix-Assisted Laser Desorption Ionization Time-of-Flight Mass Spectrometry and ClinPro Tools as a Rapid Tool for Typing Streptococcus pyogenes. Open Forum. Infect. Dis..

[62] Zhang T., Ding J., Rao X., Yu J., Chu M., Ren W., Wang L., Xue W. (2015). Analysis of methicillin-resistant Staphylococcus aureus major clonal lineages by Matrix-Assisted Laser Desorption Ionization-Time of Flight Mass Spectrometry (MALDI-TOF MS). J. Microbiol. Methods.

[63] Wang H.-Y., Lien F., Liu T.-P., Chen C.-H., Chen C.-J., Lu J.-J. (2018). Application of a MALDI-TOF analysis platform (ClinProTools) for rapid and preliminary report of MRSA sequence types in Taiwan. PeerJ.

[64] Shan W., Li J., Fang Y., Wang X., Gu D., Zhang R. (2016). Rapid Identification of Methicillin-Resistant Staphylococcus aureus (MRSA) by the Vitek MS Saramis system. Curr. Microbiol..

[65] Kehrmann J., Schoerding A.K., Murali R., Wessel S., Koehling H.L., Mosel F., Buer J. (2016). Performance of Vitek MS in identifying nontuberculous mycobacteria from MGIT liquid medium and Lowenstein-Jensen solid medium. Diagn. Microbiol. Infect. Dis..

[66] Cox C.R., Harris R.M. (2021). Mass Spectrometry and Microbial Diagnostics in the Clinical Laboratory. Clin. Lab. Med..

[67] Dortet L., Bonnin R.A., Pennisi I., Gauthier L., Jousset A.B., Dabos L., Furniss R.C.D., Mavridou D.A.I., Bogaerts P., Glupczynski Y. (2018). Rapid detection and discrimination of chromosome- and MCR-plasmid-mediated resistance to polymyxins by MALDI-TOF MS in Escherichia coli: The MALDIxin test. J. Antimicrob. Chemother..

[68] Dortet L., Potron A., Bonnin R.A., Plesiat P., Naas T., Filloux A., Larrouy-Maumus G. (2018). Rapid detection of colistin resistance in Acinetobacter baumannii using MALDI-TOF-based lipidomics on intact bacteria. Sci. Rep..

[69] Ryzhov V., Hathout Y., Fenselau C. (2000). Rapid characterization of spores of Bacillus cereus group bacteria by matrix-assisted laser desorption-ionization time-of-flight mass spectrometry. Appl. Environ. Microbiol..

[70] Giebel R.A., Fredenberg W., Sandrin T.R. (2008). Characterization of environmental isolates of Enterococcus spp. by matrix-assisted laser desorption/ionization time-of-flight mass spectrometry. Water Res..

[71] Valcárcel Cases M., Gómez Hens A. (2003). Hibridación Instrumental en Técnicas Analíticas de Separación.

[72] Perez-Riverol Y., Xu Q.W., Wang R., Uszkoreit J., Griss J., Sanchez A., Reisinger F., Csordas A., Ternent T., Del-Toro N. (2016). PRIDE Inspector Toolsuite: Moving Toward a Universal Visualization Tool for Proteomics Data Standard Formats and Quality Assessment of ProteomeXchange Datasets. Mol. Cell. Proteom. MCP.

[73] Uszkoreit J., Plohnke N., Rexroth S., Marcus K., Eisenacher M. (2014). The bacterial proteogenomic pipeline. BMC Genom..

[74] Chong Y.K., Ho C.C., Leung S.Y., Lau S.K.P., Woo P.C.Y. (2018). Clinical Mass Spectrometry in the Bioinformatics Era: A Hitchhiker’s Guide. Comput. Struct. Biotechnol. J..

[75] Mantini D., Petrucci F., Pieragostino D., Del Boccio P., Sacchetta P., Candiano G., Ghiggeri G.M., Lugaresi A., Federici G., Di Ilio C. (2010). A computational platform for MALDI-TOF mass spectrometry data: Application to serum and plasma samples. J. Proteom..

[76] Nahnsen S., Bielow C., Reinert K., Kohlbacher O. (2013). Tools for label-free peptide quantification. Mol. Cell. Proteom. MCP.

[77] Niyompanich S., Srisanga K., Jaresitthikunchai J., Roytrakul S., Tungpradabkul S. (2015). Utilization of Whole-Cell MALDI-TOF Mass Spectrometry to Differentiate Burkholderia pseudomallei Wild-Type and Constructed Mutants. PLoS ONE.

[78] Worley B., Powers R. (2013). Multivariate Analysis in Metabolomics. Curr. Metab..

[79] Santos T., Capelo J.L., Santos H.M., Oliveira I., Marinho C., Goncalves A., Araujo J.E., Poeta P., Igrejas G. (2015). Use of MALDI-TOF mass spectrometry fingerprinting to characterize Enterococcus spp. and Escherichia coli isolates. J. Proteom..

[80] Landgrebe J., Wurst W., Welzl G. (2002). Permutation-validated principal components analysis of microarray data. Genome Biol..

[81] Werth M.T., Halouska S., Shortridge M.D., Zhang B., Powers R. (2010). Analysis of metabolomic PCA data using tree diagrams. Anal. Biochem..

[82] Gibb S., Strimmer K. (2012). MALDIquant: A versatile R package for the analysis of mass spectrometry data. Bioinformatics.

[83] Lopez-Fernandez H., Santos H.M., Capelo J.L., Fdez-Riverola F., Glez-Pena D., Reboiro-Jato M. (2015). Mass-Up: An all-in-one open software application for MALDI-TOF mass spectrometry knowledge discovery. BMC Bioinform..

[84] Montoro Bustos A.R., Ruiz Encinar J., Sanz-Medel A. (2013). Mass spectrometry for the characterisation of nanoparticles. Anal. Bioanal. Chem..

[85] Torres-Sangiao E., Holban A.M., Gestal M.C. (2016). Advanced Nanobiomaterials: Vaccines, Diagnosis and Treatment of Infectious Diseases. Molecules.

[86] Torres Sangiao E., Holban A.M., Gestal M.C. (2019). Applications of Nanodiamonds in the Detection and Therapy of Infectious Diseases. Materials.

[87] Yang Y., Long C.L., Li H.P., Wang Q., Yang Z.G. (2016). Analysis of silver and gold nanoparticles in environmental water using single particle-inductively coupled plasma-mass spectrometry. Sci. Total Environ..

[88] Lopez-Cortes R., Formigo J., Reboiro-Jato M., Fdez-Riverola F., Blanco F.J., Lodeiro C., Oliveira E., Capelo J.L., Santos H.M. (2016). A methodological approach based on gold-nanoparticles followed by matrix assisted laser desorption ionization time of flight mass spectrometry for the analysis of urine profiling of knee osteoarthritis. Talanta.

[89] Shrivas K., Wu H.F. (2008). Applications of silver nanoparticles capped with different functional groups as the matrix and affinity probes in surface-assisted laser desorption/ionization time-of-flight and atmospheric pressure matrix-assisted laser desorption/ionization ion trap mass spectrometry for rapid analysis of sulfur drugs and biothiols in human urine. Rapid Commun. Mass Spectrom. RCM.

[90] Chou T.C., Hsu W., Wang C.H., Chen Y.J., Fang J.M. (2011). Rapid and specific influenza virus detection by functionalized magnetic nanoparticles and mass spectrometry. J. Nanobiotechnology.

[91] Miotto G., Magro M., Terzo M., Zaccarin M., Da Dalt L., Bonaiuto E., Baratella D., Gabai G., Vianello F. (2016). Protein corona as a proteome fingerprint: The example of hidden biomarkers for cow mastitis. Colloids Surf. B Biointerfaces.

[92] Baker M.J., Trevisan J., Bassan P., Bhargava R., Butler H.J., Dorling K.M., Fielden P.R., Fogarty S.W., Fullwood N.J., Heys K.A. (2014). Using Fourier transform IR spectroscopy to analyze biological materials. Nat. Protoc..

[93] Vatanshenassan M., Boekhout T., Mauder N., Robert V., Maier T., Meis J.F., Berman J., Then E., Kostrzewa M., Hagen F. (2020). Evaluation of Microsatellite Typing, ITS Sequencing, AFLP Fingerprinting, MALDI-TOF MS, and Fourier-Transform Infrared Spectroscopy Analysis of Candida auris. J. Fungi.

[94] Naumann D., Helm D., Labischinski H. (1991). Microbiological characterizations by FT-IR spectroscopy. Nature.

[95] Martak D., Valot B., Sauget M., Cholley P., Thouverez M., Bertrand X., Hocquet D. (2019). Fourier-Transform InfraRed Spectroscopy Can Quickly Type Gram-Negative Bacilli Responsible for Hospital Outbreaks. Front. Microbiol..

[96] Burckhardt I., Sebastian K., Mauder N., Kostrzewa M., Burckhardt F., Zimmermann S. (2019). Analysis of Streptococcus pneumoniae using Fourier-transformed infrared spectroscopy allows prediction of capsular serotype. Eur. J. Clin. Microbiol. Infect. Dis..

[97] Cordovana M., Mauder N., Kostrzewa M., Wille A., Rojak S., Hagen R.M., Ambretti S., Pongolini S., Soliani L., Justesen U.S. (2021). Classification of Salmonella enterica of the (Para-)Typhoid Fever Group by Fourier-Transform Infrared (FTIR) Spectroscopy. Microorganisms.

[98] Alexandrov T. (2012). MALDI imaging mass spectrometry: Statistical data analysis and current computational challenges. BMC Bioinform..

[99] Caprioli R.M., Farmer T.B., Gile J. (1997). Molecular imaging of biological samples: Localization of peptides and proteins using MALDI-TOF MS. Anal. Chem..

[100] Attia A.S., Schroeder K.A., Seeley E.H., Wilson K.J., Hammer N.D., Colvin D.C., Manier M.L., Nicklay J.J., Rose K.L., Gore J.C. (2012). Monitoring the inflammatory response to infection through the integration of MALDI IMS and MRI. Cell Host Microbe.

[101] Watrous J.D., Dorrestein P.C. (2011). Imaging mass spectrometry in microbiology. Nat. Rev. Microbiol..

[102] Chaurand P., Cornett D.S., Angel P.M., Caprioli R.M. (2011). From whole-body sections down to cellular level, multiscale imaging of phospholipids by MALDI mass spectrometry. Mol. Cell. Proteom. MCP.

[103] Schwamborn K., Krieg R.C., Uhlig S., Ikenberg H., Wellmann A. (2011). MALDI imaging as a specific diagnostic tool for routine cervical cytology specimens. Int. J. Mol. Med..

[104] Yi X., Li J., Yu S., Zhang A., Xu J., Yi J., Zou J., Nie X., Huang J., Wang J. (2011). A new PCR-based mass spectrometry system for high-risk HPV, part I: Methods. Am. J. Clin. Pathol..

[105] Cricca M., Marasco E., Alessandrini F., Fazio C., Prossomariti A., Savini C., Venturoli S., Chieco P., De Carolis S., Bonafe M. (2015). High-throughput genotyping of high-risk Human Papillomavirus by MALDI-TOF Mass Spectrometry-based method. New Microbiol..

[106] Calderaro A., Arcangeletti M.C., Rodighiero I., Buttrini M., Gorrini C., Motta F., Germini D., Medici M.C., Chezzi C., De Conto F. (2014). Matrix-assisted laser desorption/ionization time-of-flight (MALDI-TOF) mass spectrometry applied to virus identification. Sci. Rep..

[107] Zhang C., Xiao Y., Du J., Ren L., Wang J., Peng J., Jin Q. (2015). Application of Multiplex PCR Coupled with Matrix-Assisted Laser Desorption Ionization-Time of Flight Analysis for Simultaneous Detection of 21 Common Respiratory Viruses. J. Clin. Microbiol..

[108] Downard K.M. (2013). Proteotyping for the rapid identification of influenza virus and other biopathogens. Chem. Soc. Rev..

[109] Zurcher S., Mooser C., Luthi A.U., Muhlemann K., Barbani M.T., Mohacsi P., Garzoni C., Gorgievski-Hrisoho M., Schaller A., Flatz L. (2012). Sensitive and rapid detection of ganciclovir resistance by PCR based MALDI-TOF analysis. J. Clin. Virol..

[110] Nachtigall F.M., Pereira A., Trofymchuk O.S., Santos L.S. (2020). Detection of SARS-CoV-2 in nasal swabs using MALDI-MS. Nat. Biotechnol..

[111] Calderaro A., De Conto F., Buttrini M., Piccolo G., Montecchini S., Maccari C., Martinelli M., Di Maio A., Ferraglia F., Pinardi F. (2021). Human respiratory viruses, including SARS-CoV-2, circulating in the winter season 2019–2020 in Parma, Northern Italy. Int. J. Infect. Dis..

[112] Milewska A., Ner-Kluza J., Dabrowska A., Bodzon-Kulakowska A., Pyrc K., Suder P. (2020). MASS SPECTROMETRY IN VIROLOGICAL SCIENCES. Mass Spectrom. Rev..

[113] Calderaro A., Arcangeletti M.C., Rodighiero I., Buttrini M., Montecchini S., Vasile Simone R., Medici M.C., Chezzi C., De Conto F. (2016). Identification of different respiratory viruses, after a cell culture step, by matrix assisted laser desorption/ionization time of flight mass spectrometry (MALDI-TOF MS). Sci. Rep..

[114] Rybicka M., Miłosz E., Bielawski K.P. (2021). Superiority of MALDI-TOF Mass Spectrometry over Real-Time PCR for SARS-CoV-2 RNA Detection. Viruses.

[115] Hernandez M.M., Banu R., Shrestha P., Patel A., Chen F., Cao L., Fabre S., Tan J., Lopez H., Chiu N. (2021). RT-PCR/MALDI-TOF mass spectrometry-based detection of SARS-CoV-2 in saliva specimens. J. Med. Virol..

[116] Iles R.K., Zmuidinaite R., Iles J.K., Carnell G., Sampson A., Heeney J.L. (2020). Development of a Clinical MALDI-ToF Mass Spectrometry Assay for SARS-CoV-2: Rational Design and Multi-Disciplinary Team Work. Diagnostics.

[117] Cartelle Gestal M., Dedloff M.R., Torres-Sangiao E. (2019). Computational Health Engineering Applied to Model Infectious Diseases and Antimicrobial Resistance Spread. Appl. Sci..

[118] Tran N.K., Howard T., Walsh R., Pepper J., Loegering J., Phinney B., Salemi M.R., Rashidi H.H. (2021). Novel application of automated machine learning with MALDI-TOF-MS for rapid high-throughput screening of COVID-19: A proof of concept. Sci. Rep..

[119] Hrabak J., Chudackova E., Walkova R. (2013). Matrix-assisted laser desorption ionization-time of flight (maldi-tof) mass spectrometry for detection of antibiotic resistance mechanisms: From research to routine diagnosis. Clin. Microbiol. Rev..

[120] Machen A., Drake T., Wang Y.F. (2014). Same day identification and full panel antimicrobial susceptibility testing of bacteria from positive blood culture bottles made possible by a combined lysis-filtration method with MALDI-TOF VITEK mass spectrometry and the VITEK2 system. PLoS ONE.

[121] Nix I.D., Idelevich E.A., Storck L.M., Sparbier K., Drews O., Kostrzewa M., Becker K. (2020). Detection of Methicillin Resistance in Staphylococcus aureus From Agar Cultures and Directly From Positive Blood Cultures Using MALDI-TOF Mass Spectrometry-Based Direct-on-Target Microdroplet Growth Assay. Front. Microbiol..

[122] Correa-Martinez C.L., Idelevich E.A., Sparbier K., Kuczius T., Kostrzewa M., Becker K. (2020). Development of a MALDI-TOF MS-based screening panel for accelerated differential detection of carbapenemases in Enterobacterales using the direct-on-target microdroplet growth assay. Sci. Rep..

[123] Correa-Martinez C.L., Idelevich E.A., Sparbier K., Kostrzewa M., Becker K. (2019). Rapid Detection of Extended-Spectrum beta-Lactamases (ESBL) and AmpC beta-Lactamases in Enterobacterales: Development of a Screening Panel Using the MALDI-TOF MS-Based Direct-on-Target Microdroplet Growth Assay. Front. Microbiol..

[124] Idelevich E.A., Becker K. (2021). MALDI-TOF mass spectrometry for antimicrobial susceptibility testing. J. Clin. Microbiol..

[125] Boyle J., Rovira H., Cavnor C., Burdick D., Killcoyne S., Shmulevich I. (2009). Adaptable data management for systems biology investigations. BMC Bioinform..

[126] Morris J.H., Knudsen G.M., Verschueren E., Johnson J.R., Cimermancic P., Greninger A.L., Pico A.R. (2014). Affinity purification-mass spectrometry and network analysis to understand protein-protein interactions. Nat. Protoc..

[127] Kshirsagar M., Carbonell J., Klein-Seetharaman J. (2013). Multitask learning for host-pathogen protein interactions. Bioinformatics.

[128] Szklarczyk D., Franceschini A., Wyder S., Forslund K., Heller D., Huerta-Cepas J., Simonovic M., Roth A., Santos A., Tsafou K.P. (2015). STRING v10: Protein-protein interaction networks, integrated over the tree of life. Nucleic Acids Res..

[129] Fuchs S., Zuhlke D., Pane-Farre J., Kusch H., Wolf C., Reiss S., Binh L.T.N., Albrecht D., Riedel K., Hecker M. (2013). Aureolib—A proteome signature library: Towards an understanding of staphylococcus aureus pathophysiology. PLoS ONE.

[130] Oughtred R., Chatr-Aryamontri A., Breitkreutz B.J., Chang C.S., Rust J.M., Theesfeld C.L., Heinicke S., Breitkreutz A., Chen D., Hirschman J. (2016). BioGRID: A Resource for Studying Biological Interactions in Yeast. Cold Spring Harb. Protoc..

[131] Stark C., Breitkreutz B.J., Reguly T., Boucher L., Breitkreutz A., Tyers M. (2006). BioGRID: A general repository for interaction datasets. Nucleic Acids Res..

[132] Shannon P., Markiel A., Ozier O., Baliga N.S., Wang J.T., Ramage D., Amin N., Schwikowski B., Ideker T. (2003). Cytoscape: A software environment for integrated models of biomolecular interaction networks. Genome Res..

[133] Smoot M.E., Ono K., Ruscheinski J., Wang P.L., Ideker T. (2011). Cytoscape 2.8: New features for data integration and network visualization. Bioinformatics.

[134] Vaudel M., Burkhart J.M., Zahedi R.P., Oveland E., Berven F.S., Sickmann A., Martens L., Barsnes H. (2015). PeptideShaker enables reanalysis of MS-derived proteomics data sets. Nat. Biotechnol..

[135] Desiere F., Deutsch E.W., King N.L., Nesvizhskii A.I., Mallick P., Eng J., Chen S., Eddes J., Loevenich S.N., Aebersold R. (2006). The PeptideAtlas project. Nucleic Acids Res..

[136] Fabregat A., Sidiropoulos K., Garapati P., Gillespie M., Hausmann K., Haw R., Jassal B., Jupe S., Korninger F., McKay S. (2016). The Reactome pathway Knowledgebase. Nucleic Acids Res..

[137] Snel B., Lehmann G., Bork P., Huynen M.A. (2000). STRING: A web-server to retrieve and display the repeatedly occurring neighbourhood of a gene. Nucleic Acids Res..

[138] Yoo J.I., Kim J.W., Kang G.S., Kim H.S., Yoo J.S., Lee Y.S. (2013). Prevalence of amino acid changes in the yvqF, vraSR, graSR, and tcaRAB genes from vancomycin intermediate resistant Staphylococcus aureus. J. Microbiol..

[139] McCallum N., Meier P.S., Heusser R., Berger-Bachi B. (2011). Mutational analyses of open reading frames within the vraSR operon and their roles in the cell wall stress response of Staphylococcus aureus. Antimicrob. Agents Chemother..

[140] Gardete S., Tomasz A. (2014). Mechanisms of vancomycin resistance in Staphylococcus aureus. J. Clin. Investig..

